# Three-year follow-up of epcoritamab therapy in Japanese patients with relapsed/refractory follicular lymphoma in EPCORE NHL-3

**DOI:** 10.1007/s12185-025-04139-1

**Published:** 2026-01-18

**Authors:** Koji Izutsu, Daigo Akahane, Tomomi Toubai, Toko Saito, Yuko Mishima, Tomoaki Fujisaki, Momoko Nishikori, Takahiro Kumode, Youko Suehiro, Kenji Ishitsuka, Poliana Patah, Ami Takahashi, Barbara D’Angelo Månsson, Elena Favaro, Noriko Fukuhara

**Affiliations:** 1 Department of Hematology, National Cancer Center Hospital, Tsukiji 5-1-1, Chuo-ku, Tokyo, 104-0045 Japan; 2 Department of Hematology, Tokyo Medical University, Tokyo, Japan; 3 Department of Internal Medicine III, Division of Hematology and Cell Therapy, Faculty of Medicine, Yamagata University, Yamagata, Japan; 4 Department of Hematology and Cell Therapy, Aichi Cancer Center, Nagoya, Japan; 5 Department of Hematology Oncology, Cancer Institute Hospital, Japanese Foundation for Cancer Research, Tokyo, Japan; 6 Department of Hematology, Japan Red Cross Society, Matsuyama Red Cross Hospital, Matsuyama, Japan; 7 Department of Hematology, Graduate School of Medicine, Kyoto University, Kyoto, Japan; 8 Department of Hematology and Rheumatology, Kindai University, Osaka, Japan; 9 Department of Hematology and Cell Therapy, National Hospital Organization Kyushu Cancer Center, Fukuoka, Japan; 10 Department of Hematology and Rheumatology, Kagoshima University, Kagoshima, Japan; 11 AbbVie, North Chicago, IL USA; 12 Genmab, Tokyo, Japan; 13 Genmab, Copenhagen, Denmark; 14 Department of Hematology, Tohoku University, Sendai, Japan

**Keywords:** Bispecific antibodies, Clinical trial, Follicular lymphoma, Non-Hodgkin lymphoma

## Abstract

**Background:**

The CD3xCD20 bispecific antibody epcoritamab demonstrated deep, durable responses with manageable safety in relapsed/refractory (R/R) follicular lymphoma (FL) in EPCORE NHL-3 (phase 1/2; NCT04542824). This analysis evaluated 3-year follow-up outcomes in Japanese patients.

**Methods:**

Adults with CD20 + FL and ≥ 2 prior lines of therapy (LOTs) received subcutaneous epcoritamab (0.16/0.8-mg step-up doses; 48-mg full doses) until disease progression or death. The primary endpoint was overall response rate (ORR).

**Results:**

Twenty-one patients received epcoritamab (median age: 65 years; median prior LOTs: 4; 57.1% double refractory; 57.1% progressed within 24 months of any first-line treatment). At a median follow-up of 35 months, the ORR was 95.2% and the complete response (CR) rate was 76.2%. Median duration of response, duration of CR, progression-free survival, and overall survival were not reached. At 3 years, 66.7% and 93.3% of complete responders remained progression-free and alive, respectively. Minimal residual disease negativity was achieved in 88.9% (16/18) of evaluable patients. Common treatment-emergent adverse events included cytokine release syndrome (90.5%) and injection-site reaction (71.4%), all predominantly grade 1–2 and occurring in early treatment cycles, and none fatal.

**Conclusions:**

Epcoritamab monotherapy provided durable long-term remission with favorable safety in Japanese patients with R/R FL over 3 years of follow-up.

**Supplementary Information:**

The online version contains supplementary material available at 10.1007/s12185-025-04139-1.

## Introduction

In Japan, follicular lymphoma (FL) accounts for approximately 10–20% of all non-Hodgkin lymphoma (NHL) cases, making it the second most prevalent NHL subtype after diffuse large B-cell lymphoma (DLBCL) [1, 2]. Although typically indolent, FL remains incurable. Overall response rates (ORRs) worsen with subsequent lines of therapy (LOTs) and are reported to be approximately 70% for patients receiving third-line treatment [3]. FL also carries a 30% lifetime risk of transformation to DLBCL, which is more aggressive [3–6]. Patients with progression of disease within 24 months (POD24) of any first-line treatment and those with double-refractory disease have particularly poor prognoses [3, 7, 8]. There is no standard of care for patients with relapsed/refractory (R/R) FL, and historical approaches have comprised retreatment with rituximab- and chemoimmunotherapy-based regimens. In recent years, chimeric antigen receptor (CAR) T-cell therapy has shown promising efficacy in this setting despite notable safety concerns; however, access to CAR T-cell therapy in Japan remains limited to a small number of specialized centers [4, 9–13]. While CAR T-cell therapies have also shown efficacy in patients with R/R FL who have received multiple prior treatments, the evidence supporting their potential benefit in FL remains limited, as pivotal clinical trials reported to date were single-arm studies without a comparator arm. At present, due to the relatively short follow-up periods, it remains unclear whether CAR T-cell therapy can achieve a curative outcome in patients with R/R FL [10, 11]. There remains a need for R/R FL treatment options that balance efficacy and tolerability to ensure long-term clinical benefit.

Bispecific antibodies (BsAbs) have emerged as highly effective T-cell-engaging therapies for the management of R/R B-cell NHL, addressing a critical need in patients with few effective treatment options [4, 14]. BsAbs are an off-the-shelf alternative to CAR T-cell therapy that offer comparable efficacy with more predictable and manageable safety profiles, as well as the potential for outpatient administration and greater patient access [4].

Epcoritamab is a subcutaneous (SC) CD3xCD20 BsAb that is approved as monotherapy for patients with R/R FL after 2 prior LOTs in Japan, the United States, and Europe [15–17]. Epcoritamab is also the only SC BsAb approved for patients with R/R FL and large B-cell lymphoma (DLBCL, high-grade B-cell lymphoma, primary mediastinal large B-cell lymphoma), making it a broadly effective and accessible treatment option for patients and healthcare systems.

Epcoritamab monotherapy demonstrated deep and durable responses with a manageable safety profile in patients with R/R FL in the pivotal, phase 2, global EPCORE NHL-1 trial (NCT03625037) [14]. At a median follow-up of 17.4 months, the ORR was 82%, with 63% of patients achieving a complete response (CR) [14]. Of note, patients were heavily pretreated with a median of 3 prior LOTs, and 52% were POD24 after any first-line treatment [14]. The pivotal phase 1/2 EPCORE NHL-3 trial (NCT04542824; JapicCTI‐205,408; jRCT2080225312) was conducted in Japan among patients with R/R CD20 + B-cell NHL treated with ≥ 2 prior LOTs and showed consistent findings with those of the NHL-1 trial, where 2-year follow-up data demonstrated a high ORR and CR rate (95.2% and 76.2%, respectively) with a manageable safety profile [18]. Favorable outcomes were also observed among patients with double-refractory disease, POD24, and prior bendamustine use, further validating the efficacy and safety of epcoritamab in this setting.

This analysis extends the previous findings from EPCORE NHL-3 with 3 years of follow-up, providing the longest duration of follow-up to date for BsAb therapy in patients with R/R FL in Japan. These longer term follow-up data offer further evidence supporting the durability and robustness of epcoritamab treatment responses and long-term safety among patients with R/R FL, including heavily pretreated patients and those with challenging-to-treat disease features.

## Materials and methods

### Study design and patients

EPCORE NHL-3 is a phase 1/2, open-label, dose-escalation and -expansion study evaluating SC epcoritamab in patients with R/R mature B-cell NHL. Details on the design, methods, and primary findings from the EPCORE NHL-3 study have been reported previously [18]. Eligible patients were adults (≥ 20 years of age) with CD20 + disease and ≥ 2 prior LOTs, including ≥ 1 anti-CD20 monoclonal antibody (mAb)-containing regimen. The FL grade 1–3A expansion cohort included in this analysis received 2 step-up doses (0.16/0.8 mg), followed by 48-mg full doses of epcoritamab in 28-day cycles until progressive disease (PD) or unacceptable toxicity (once weekly in cycles [C] 1–3, once every 2 weeks in C4–9, and once every 4 weeks in C10 and thereafter). Prophylaxis for cytokine release syndrome (CRS) was administered as previously described [18]. Patients were hospitalized for ≥ 24 h after the first full dose in the expansion cohort to monitor for potential CRS.

### Endpoints and assessments

The key efficacy measure in this paper is investigator-assessed ORR per Lugano criteria [19]. Response was evaluated using positron emission tomography and computed tomography at 6, 12, 18, 24, 36, and 48 weeks, and every 6 months thereafter until PD. Additional efficacy endpoints included CR rate, time to response (TTR), time to CR (TTCR), duration of response (DOR), duration of CR (DOCR), progression-free survival (PFS), overall survival (OS), and minimal residual disease (MRD)-negativity rates. These response-related endpoints were investigator-assessed, similar to ORR analyses. MRD was assessed in peripheral blood mononuclear cells using the clonoSEQ® (Adaptive Biotechnologies, Seattle, WA, USA) next-generation sequencing assay with a cutoff of 10^−6^ (tumor clones detected per 1 million nucleated cells).

Safety assessments included rates and severity of adverse events (AEs), laboratory abnormalities, cytokine levels, and immunoglobulin (Ig) G levels. All AEs were graded using the National Cancer Institute Common Terminology Criteria for Adverse Events version 5.0 (NCI CTCAE v5.0) [20]. CRS and immune effector cell-associated neurotoxicity syndrome events were graded according to the American Society for Transplantation and Cellular Therapy (ASTCT) criteria, and clinical tumor lysis syndrome events were graded according to Cairo–Bishop guidelines [21, 22].

### Statistical analysis

Efficacy and safety were evaluated in the full analysis set and the safety analysis set, both of which included all patients with R/R FL grade 1–3A who had received ≥ 1 dose of epcoritamab. The MRD-evaluable set comprised all patients in the full analysis set who had ≥ 1 on-treatment MRD sample.

All outcomes were analyzed using descriptive statistics including mean, median, and standard deviation for continuous variables, and frequencies and proportions with 95% confidence intervals (CIs) for categorical variables. Time-to-event data were analyzed using the Kaplan–Meier method.

ORR was defined as the proportion of patients who achieved a best clinical response of CR or partial response (PR). Sample size calculation was performed for the primary analysis and has been reported previously [18]. The 95% CI for the ORR and CR rate was calculated using the Clopper–Pearson method. All analyses were conducted using SAS 9.4 (TS1M7) in Windows Server 2008 R2.

## Results

### Patient population

As of July 12, 2024 a total of 21 patients with R/R FL had received SC epcoritamab; the median follow-up was 35.0 months (range, 11.0–41.2). As reported previously, patients were heavily pretreated (median, 4 prior LOTs) and had high-risk features, including 57.1% with double-refractory disease and 57.1% with POD24 (Table S1) [18].

### Treatment exposure and disposition

**Fig. 1:**
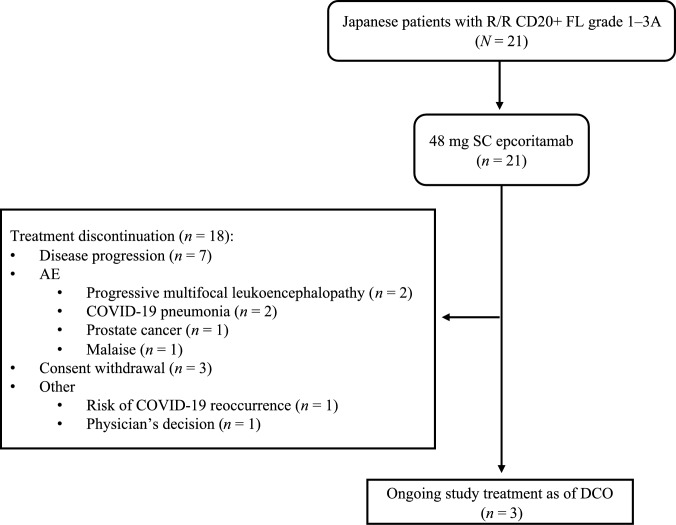
Flowchart illustrating patient enrollment and disposition in the EPCORE NHL-3 study for the R/R FL cohort as of the July 12, 2024 DCO date. *AE* adverse event, *COVID-19* coronavirus disease 2019, *DCO* data cutoff, *FL* follicular lymphoma, *R/R* relapsed/refractory, *SC* subcutaneous

Of the 21 patients who received epcoritamab, 3 (14.3%) were still on treatment at the data cutoff and 18 (85.7%) had discontinued (Fig. 1). Overall, patients received a median of 13 treatment cycles (range, 1–43), and the median duration of treatment was 11.1 months (range, 1–39). The reasons for treatment discontinuation were PD (7/18 [38.9%]), AEs (6/18 [33.3%]), consent withdrawal (3/18 [16.7%]), risk of coronavirus disease 2019 (COVID-19) recurrence (*n* = 1), and physician’s decision (*n* = 1). AEs leading to discontinuation were progressive multifocal leukoencephalopathy (PML; *n* = 2), COVID-19 pneumonia (*n* = 2), prostate cancer (*n* = 1), and malaise (*n* = 1).

### Efficacy

Consistent with the primary analysis, epcoritamab demonstrated an ORR of 95.2% (20/21; 95% CI, 76.2–99.9) and a CR rate of 76.2% (16/21; 95% CI, 52.8–91.8). Only 1 patient had a PR as of the last assessment of the primary analysis, but this patient had no subsequent scans, and therefore, there was no evidence of conversion from PR to CR with longer follow-up. Median TTR was 1.3 months (range, 1.1–1.7) and median TTCR was 1.4 months (range, 1.1–3.6), with most CRs achieved by the first 6-week assessment (Table 1).

**Table 1:** Efficacy endpoints in the overall R/R FL population

Endpoint	Patients (*N* = 21)
Median DOR, months (range)	NR (0.00 + to 37.6 +)
Median DOCR, months (range)	NR (1.3–37.6 +)
Median PFS, months (95% CI)	NR (4.0–NR)
Patients with CR^a^	NR (7.9–NR)
MRD-negative patients^b^	NR (2.1–NR)
Median OS, months (95% CI)	NR (NR–NR)
36-month OS,^c^ % (95% CI)	89.7 (64.8–97.3)
Median TTNT, months (range)	NR (3.1–39.2 +)
36-month TTNT,^c^ % (95% CI)	65.9 (41.4–82.2)
Median TTR, months (range)	1.3 (1.1–1.7)
Median TTCR, months (range)	1.4 (1.1–3.6)
MRD negativity,^d^ *n* (%)	16 (88.9)

*CI* confidence interval, *CR* complete response, *C* cycle, *D* day, *DOCR* duration of complete response, *DOR* duration of response, *MRD* minimal residual disease, *NR* not reached, *OS* overall survival, *PFS* progression-free survival, *R/R FL* relapsed/refractory follicular lymphoma, *TTCR* time to complete response, *TTNT* time to next treatment, *TTR* time to response

^a^*n* = 16

^b^By C3D1; *n* = 12

^c^Based on Kaplan–Meier estimate at 36 months

^d^Based on clonoSEQ® peripheral blood mononuclear cell assay with 10^−6^ cutoff; *n* = 18

**Fig. 2:**
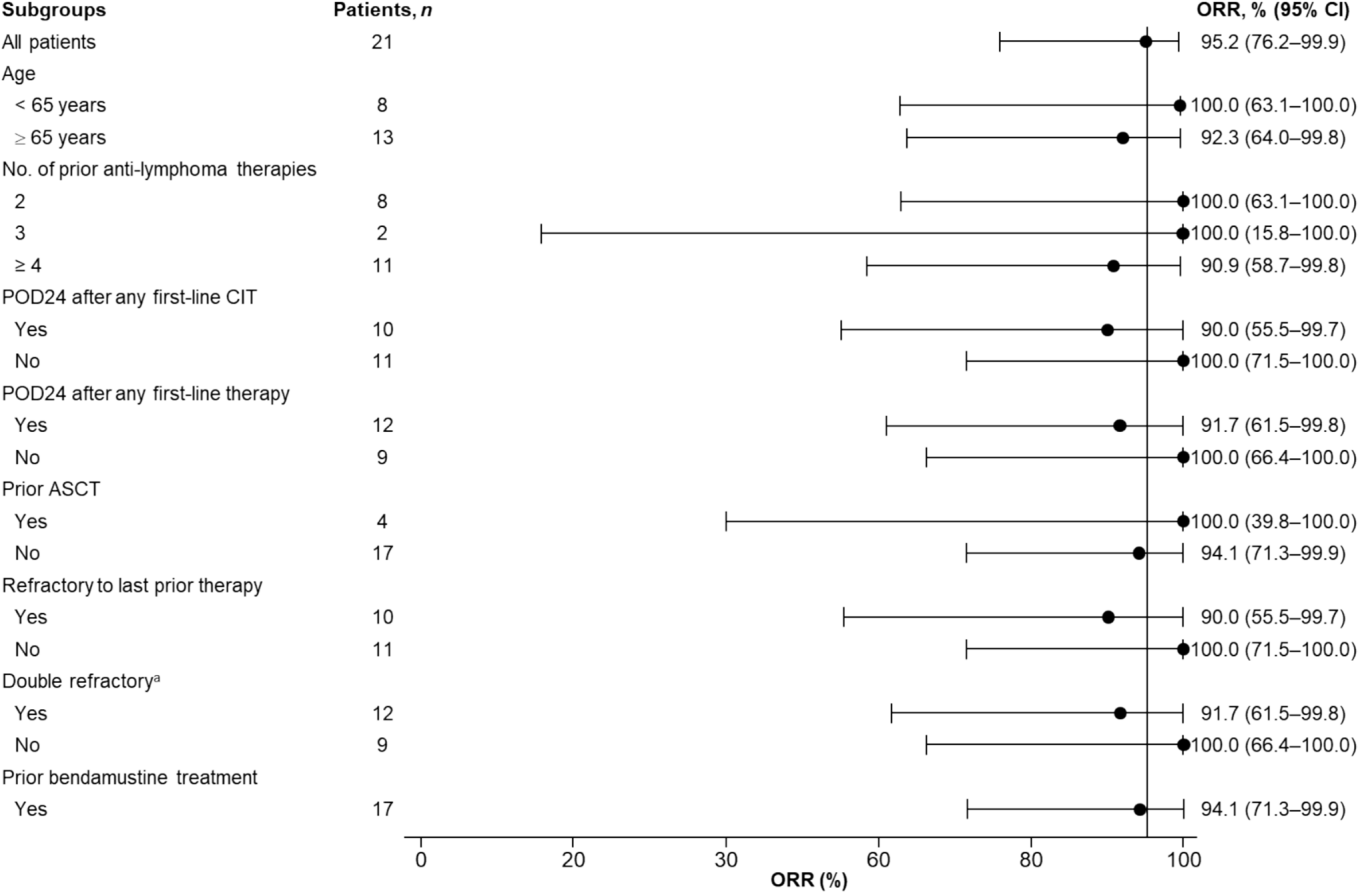
Forest plot displaying the best ORR across various clinically relevant patient subgroups based on baseline characteristics. The bars represent the percentage of patients in each response category within a 95% CI. ^a^Refractory to both an anti-CD20 mAb and an alkylating agent, regardless of whether they are in the same or different lines of treatment*. ASCT* autologous stem cell transplant, *CI* confidence interval; *CIT* chemoimmunotherapy, *mAb* monoclonal antibody, *ORR* overall response rate, *POD24* progression of disease within 24 months

High response rates (ORRs, 90–100%; Fig. 2) were also seen in patients with challenging-to-treat disease features, including patients refractory to their last LOT, those with POD24, those with double-refractory disease, patients ≥ 65 years of age, and those 65 to < 75 years of age.

**Fig. 3:**
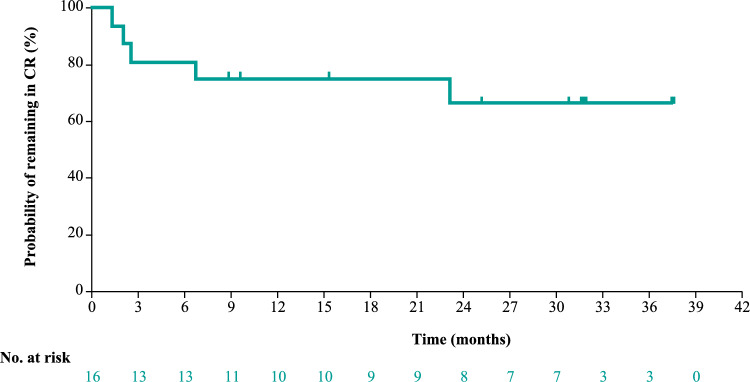
Kaplan–Meier curve for DOCR in patients with a CR (*n* = 16). The y-axis represents the probability of maintaining a CR, and the x-axis represents the time in months from the first documented CR. Tick marks indicate censored patients. The median DOCR was not reached. *CR* complete response, *DOCR* duration of complete response

**Fig. 4:**
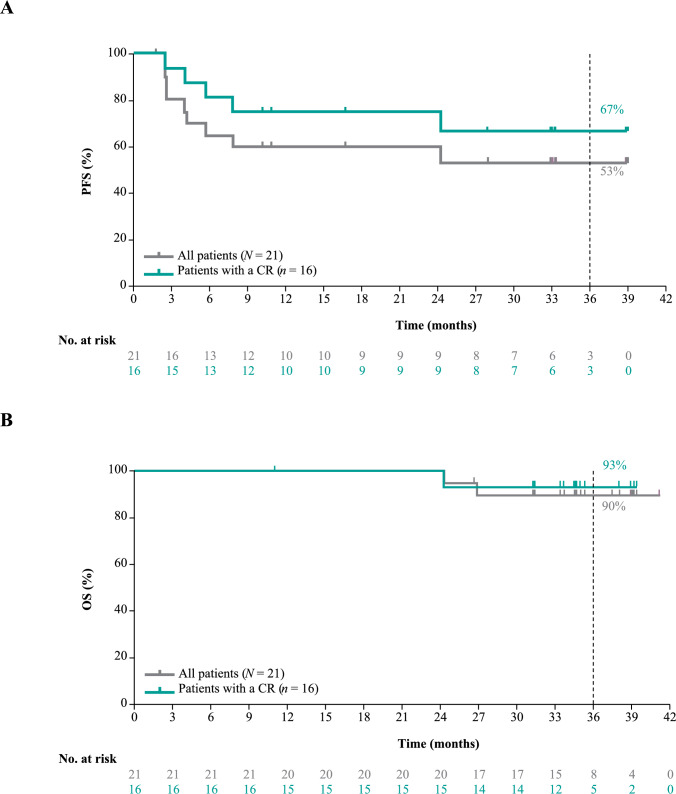
Kaplan–Meier curves illustrating PFS (A) and OS (B) for the overall patient population (*N* = 21) and for patients who achieved a CR (*n* = 16). The y-axis represents the probability of survival (PFS or OS), and the x-axis represents the time in months. Tick marks indicate censored patients. *CR* complete response*, OS* overall survival, *PFS* progression-free survival

At data cutoff, median DOR, DOCR, PFS, and OS were not reached (NR) in the overall population, with 89.7% of patients alive at 36 months (Table 1; Figs. 3 and 4). For patients achieving CR, median DOCR, PFS, and OS were also NR; at 36 months, 66.7% remained in CR, 66.7% remained progression-free, and 93.3% were alive (Fig. 4).

The majority of patients (14/21) did not proceed to the next treatment. Among the 7 (33.3%) patients who did proceed to the next treatment, 3 (14.3%) received CAR T-cell therapy and the remaining received a variety of chemoimmunotherapy. Of the 7 patients, 3 achieved a best response of CR, 3 achieved PR, and 1 had stable disease while on the next treatment. As of the data cutoff, 5 of the 7 patients were alive, 1 died, and 1 was lost to follow-up.

Of the 17/21 (81.0%) patients with prior bendamustine exposure (median time from last prior bendamustine treatment, 141 days [range, 1–214]), 13 (76.5%) achieved a CR, 3 had a PR, and 1 had stable disease while receiving epcoritamab treatment. The median PFS was 24.3 months (95% CI, 2.6–NR) and median OS was NR in this subgroup.

**Fig. 5:**
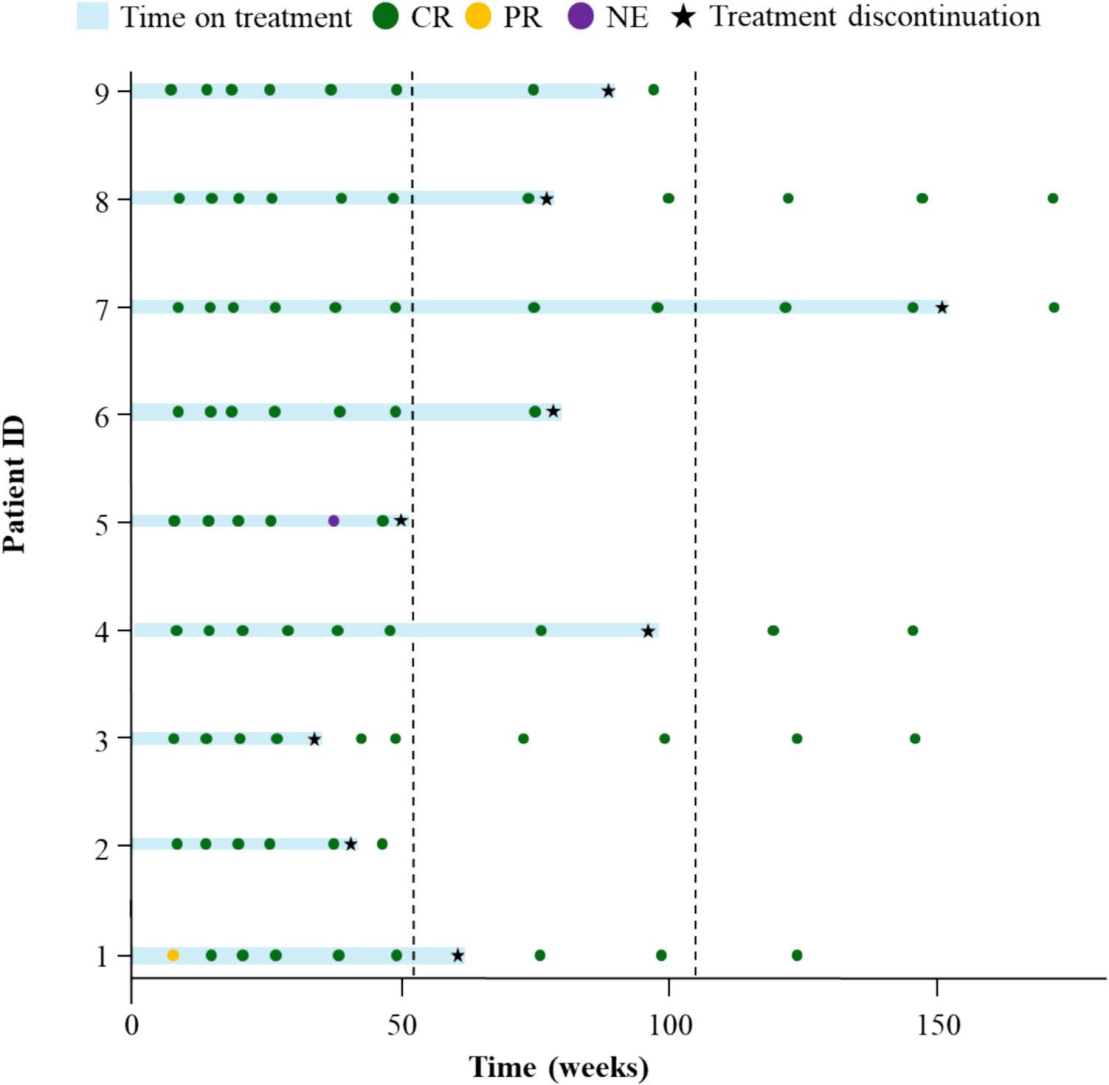
Swim lane plot detailing the treatment and response duration for patients with a CR as their last assessment who discontinued treatment for reasons other than PD (*n* = 9). An additional patient achieved CR as the best overall response; however, this patient was excluded from the figure since the last assessment prior to discontinuation was not CR. Per protocol, patients continued to receive scans if they discontinued treatment for reasons other than PD. Each bar represents an individual patient. The length of the bar indicates the time from the start of treatment, with symbols showing the best overall response and the timing of the last disease assessment. Vertical lines indicate 1- and 2-year landmarks. *CR* complete response, *DCO*, data cutoff, *NE* not evaluable, *PD* progressive disease, *PR* partial response

Among the 13 patients who achieved a CR and discontinued treatment, reasons for discontinuation were PD (*n* = 3), withdrawal by the subject (*n* = 2), and other reasons (*n* = 2: risk of recurrence of COVID-19 [*n* = 1] and investigator’s decision [*n* = 1]). The remaining 6 patients discontinued due to AEs. Nine of these patients had a CR as their last assessment and discontinued treatment for reasons other than PD (Fig. 5). Of these 9 patients (median time on treatment, 16.7 months [range, 7.7–34.6]), 7 had a documented CR after discontinuing epcoritamab without initiating subsequent therapy, and 2 did not have a post-discontinuation follow-up. All 7 patients remained in CR at the time of their last imaging evaluation (prior to data cutoff), with a median time from treatment discontinuation to this assessment of 12.3 months (range, 1.0–25.4).

Of the 18/21 (85.7%) MRD-evaluable patients, 16 (88.9%) achieved MRD negativity. In patients who were MRD-negative by the landmark C3 day (D) 1 (*n* = 12) time point, median PFS was NR (95% CI, 2.1–NR) as of data cutoff (Table 1).

### Safety

The safety and tolerability outcomes observed in this 3-year follow-up analysis were largely consistent with those observed in the primary 2-year analysis [18]. CRS (19/21 [90.5%]), injection-site reaction (15/21 [71.4%]), neutropenia (8/21 [38.1%]), rash (8/21 [38.1%]), increased alanine aminotransferase (6/21 [28.6%]), increased aspartate aminotransferase (6/21 [28.6%]), and COVID-19 (6/21 [28.6%]) remained the most reported treatment-emergent adverse events (TEAEs) in ≥ 20% of patients (Table 2). Of the 15/21 (71.4%) patients with grade 3/4 TEAEs as of the current data cutoff, 2 patients experienced new events, all unrelated to epcoritamab treatment, since the time of primary analysis: 1 patient with grade 3 events of stomatitis (not recovered/not resolved at the time of data cutoff) and decreased white blood cells (recovered/resolved) and 1 patient with a grade 3 event of upper respiratory tract infection (recovered/resolved). TEAEs led to dose delays in 14/21 (66.7%) patients. With an additional 13.8 months of follow-up since the primary report, 2 new TEAEs leading to treatment discontinuation were observed (both COVID-19 and related to epcoritamab treatment). Serious TEAEs were reported in 13/21 (61.9%) patients; 1 new serious TEAE of stomatitis, unrelated to epcoritamab, occurred since the primary analysis. No fatal TEAEs occurred during the trial.

**Table 2:** TEAE summary and most common TEAEs occurring in ≥ 20% of patients

Event^a^	Patients (*N* = 21)
Grade 3/4 TEAEs	15 (71.4)
Serious TEAE	13 (61.9)
Serious infections	10 (47.6)
Fatal TEAE	0
TEAEs leading to dose delay	14 (66.7)
TEAEs leading to treatment discontinuation	6 (28.6)
TEAEs of interest	
CRS	19 (90.5)
ICANS	0
CTLS	0
COVID-19^b^	6 (28.6)
Febrile neutropenia	1 (4.8)

TEAEs occurring in ≥ 20% of patients	Grade 1/2	Grade 3	Grade 4	Total
CRS	18 (85.7)	1 (4.8)	0	19 (90.5)
Injection-site reaction^c^	15 (71.4)	0	0	15 (71.4)
Neutropenia^d^	2 (9.5)	2 (9.5)	4 (19.0)	8 (38.1)
Rash	8 (38.1)	0	0	8 (38.1)
Increased ALT	2 (9.5)	3 (14.3)	1 (4.8)	6 (28.6)
Increased AST	5 (23.8)	1 (4.8)	0	6 (28.6)
COVID-19^b^	2 (9.5)	4 (19.0)	0	6 (28.6)
Constipation	4 (19.0)	1 (4.8)	0	5 (23.8)
Insomnia	5 (23.8)	0	0	5 (23.8)
Pyrexia	5 (23.8)	0	0	5 (23.8)

*ALT* alanine aminotransferase, *AST* aspartate aminotransferase, *COVID-19* coronavirus disease 2019, *CRS* cytokine release syndrome, *CTLS* clinical tumor lysis syndrome, *ICANS* immune effector cell-associated neurotoxicity syndrome, *TEAE* treatment-emergent adverse event

^a^All data are reported as *n* (%)

^b^Combined term includes COVID-19 and COVID-19 pneumonia

^c^Combined term includes injection-site reaction and erythema

^d^Combined term includes neutropenia and neutrophil count decreased

No new CRS events were observed since the primary analysis with longer follow-up. As previously reported, CRS occurred in 90.5% of patients; events were predominantly grade 1–2, manageable, and all resolved without leading to treatment discontinuation [18]. No immune effector cell-associated neurotoxicity syndrome or clinical tumor lysis syndrome events were reported during the trial.

The EPCORE NHL-3 trial was conducted during the peak of the COVID-19 pandemic and during the time period when the highly infectious Omicron variant and subsequent variants were prevalent. COVID-19 occurred in 6/21 (28.6%) patients over the course of the study; 2 of these patients experienced new events of grade 2/3 COVID-19 after the primary analysis, both of whom discontinued treatment. At data cutoff, 5/6 patients had COVID-19 that had resolved or were recovering/resolving. Maintenance of response was preserved, irrespective of COVID-19 and any resulting deviations in treatment adherence; 6 patients had at least one follow-up scan after COVID-19 and all remained in response (4 CRs and 1 PR who converted to CR after treatment was resumed post COVID-19) except 1 patient who had PD before COVID-19.

**Fig. 6:**
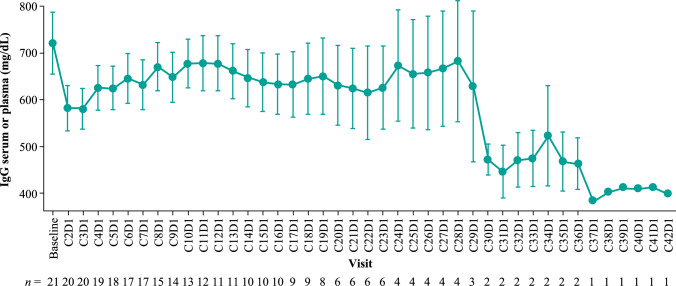
Line graph showing the mean IgG levels (mg/dL) in patients over the course of treatment, from baseline through C28D1. The points represent the mean IgG level at each time point, and the error bars indicate the standard error of the mean. The numbers below the x-axis denote the number of patients with available data at each assessment. *C* cycle,* D* day, *IgG* immunoglobulin G

Seven patients reported grade ≥ 3 infections, with only 1 grade 3 infection of upper respiratory tract infection reported beyond 2 years of treatment. Grade ≥ 3 infections were mostly viral and respiratory in nature, with no fungal infections or cytomegalovirus reactivations observed. A total of 9 patients received concomitant Ig therapy as of the data cutoff, 2 of whom were new recipients since the primary analysis. Six patients received granulocyte colony-stimulating factor (G-CSF) treatment for grade 3/4 neutropenia during the study; 1 of these patients also experienced an event of grade 3 febrile neutropenia that required treatment with G-CSF and the event resolved. After an initial decline from baseline, a trend to recovery of IgG levels was observed as early as C4D1 and remained stable through C28D1 (Fig. 6).

## Discussion

These longer term findings from the EPCORE NHL-3 study are consistent with those observed in the 2-year primary analysis, further supporting the long-term efficacy benefit and sustained remission rates achievable with epcoritamab in patients with R/R FL [18]. These findings also align with the deep and durable responses observed at 3 years among patients with DLBCL in the EPCORE NHL-3 expansion cohort, supporting the potential for long-term disease control with epcoritamab across NHL histologies [23]. The deep and durable responses observed with epcoritamab in these R/R FL patients with challenging-to-treat disease features are particularly encouraging since treatment efficacy in this population has generally appeared to wane substantially with subsequent LOTs.

These findings are consistent with prior analyses demonstrating deep and durable responses with manageable safety at 3 years of follow-up in patients with R/R FL receiving epcoritamab in Japan. The majority of patients achieved a deep and durable response, demonstrated by an ORR of 95.2% and a CR rate of 76.2%. High response and CR rates were also observed across subgroups of patients with challenging-to-treat disease features, including patients with POD24 and those with double-refractory disease, who achieved ORRs of 92% and 92%, respectively.

As of the July 12, 2024 cutoff date, the median PFS and OS were NR, with two-thirds (66.7%) of patients who achieved CR remaining progression-free and nearly all (93.3%) alive at 3 years. Despite most patients having challenging-to-treat disease features, MRD-negativity rates were 88.9%, and patients maintained their responses even after discontinuing epcoritamab treatment. This finding offers novel insight into treatment durability, as 7/9 (77.8%) patients who discontinued treatment for reasons other than PD maintained their CR for a median of 12.3 months post-discontinuation, thus demonstrating that patients can derive long-term benefit even when not treated until progression. Bendamustine plus rituximab is a common treatment option for patients with R/R FL in Japan among several guideline-supported regimens, as there is no single standard of care in this setting [24]. In this study, the majority of patients who had received prior bendamustine treatment (most commonly > 1 year prior to starting epcoritamab) achieved a CR with epcoritamab (76.5%, *n* = 13/17), suggesting that prior exposure to bendamustine did not adversely impact the efficacy benefit of epcoritamab. This finding is supported by recent data, which indicate that bendamustine exposure within 24 months of receipt of BsAbs does not appear to negatively impact key efficacy outcomes (ORR, CR rate, and PFS) in patients with R/R FL [25]. This is particularly important in the context of impaired immune function and reduced efficacy of immune-based therapies, such as CAR T-cell treatments, which are associated with prior bendamustine treatment [4, 13]. Epcoritamab may provide an effective, off-the-shelf, chemotherapy-free treatment option, even when managing more complex clinical factors.

The SC administration of epcoritamab, together with its dual approved indications in Japan, provides unique clinical value compared with other BsAbs that are administered intravenously and approved only for patients with R/R FL. Although the sample size in this trial was relatively small and informal cross-trial comparisons should be interpreted with caution due to differences in study design and patient populations, the high CR rate observed with epcoritamab in this study is encouraging in the context of efficacy findings for other BsAbs in R/R FL [26].

The long-term safety profile of epcoritamab was manageable and consistent with that observed in the EPCORE NHL-1 trial [14] and the EPCORE NHL-3 primary analysis with 2 years of follow-up [18]. No new safety signals were detected during the additional year of follow-up. Most CRS events were low grade. Dose optimization strategies from other epcoritamab studies, such as the EPCORE NHL-1 FL optimization cohort, which included an additional step-up dose (including dexamethasone as the preferred steroid and adequate hydration), have shown significantly lower rates and severity of CRS, a learning incorporated into the approved dosing regimen for enhanced mitigation [14].

The overall incidence of grade ≥ 3 infections was largely consistent with the primary analysis. Hematologic malignancies and associated treatments, including CD20-directed therapies, such as rituximab and other BsAbs, have been associated with an increased risk and severity of infections, including COVID-19. The most common infection observed in this study was COVID-19 (6 [29%] patients; 2 had grade 2 infection and 4 had grade 3 infection). Two of these COVID-19 cases were experienced after the primary analysis during the additional 13.8 months of follow-up. It should be noted that the EPCORE NHL-3 trial was conducted during the peak of the COVID-19 pandemic while the spread of the highly infectious Omicron variant and subsequent variants were prevalent. The high-risk clinical characteristics of patients participating in this trial, including age ≥ 65 years (62% of this study’s population), are established risk factors for severe COVID-19 outcomes. Despite this, all 6 patients with COVID-19 had a CR as their best overall response, and the COVID-19 events were manageable, with 5 out of 6 recovered/resolved at the time of data cutoff for this analysis. Concomitant Ig and G-CSF therapy were used in 9 and 6 patients, respectively. IgG levels tended to recover by C4D1. Infection rates and recovery of IgG levels during epcoritamab treatment were consistent with previous trials and manageable, reinforcing the potential for a long-term treatment course without compromising humoral immunity [27]. Two patients experienced PML leading to treatment discontinuation. It is important to note that PML is a known, rare complication associated with other B-cell-depleting therapies, including rituximab and obinutuzumab, and is a boxed warning for these agents [28, 29]. Patients in this trial were heavily pretreated across multiple lines of therapy, including with lymphodepleting agents that have been associated with PML [28–31]. While the development of PML in this refractory patient population is likely multifactorial, the potential contribution of an altered immune state from T-cell-engaging therapies like epcoritamab cannot be excluded and warrants continued investigation in larger patient populations. The overall infection risk was otherwise manageable and consistent with previous reports.

The observed long-term tolerability of epcoritamab, including the proactive management of neutropenia, may help mitigate concerns regarding infection risk and immune suppression with extended use. Taken together, these findings support the long-term use of epcoritamab as an effective and tolerable treat-to-progression regimen.

As an off-the-shelf, SC T-cell-engaging therapy that can be administered in the outpatient setting, epcoritamab offers potential access and convenience advantages for patients with R/R FL. This may be particularly important in Japan where access to CAR T-cell therapy remains limited [4, 13]. The outpatient administration of epcoritamab and its general accessibility to patients, supported by these confirmatory long-term efficacy and safety outcomes, may support its use in routine clinical practice following its recent approval for R/R FL in Japan.

## Conclusions

The longer term follow-up data from the EPCORE NHL-3 trial demonstrate consistently deep and durable responses and long-term remission at 3 years with epcoritamab treatment for patients with R/R FL, including those with difficulty remaining on a full treatment course and those with challenging-to-treat disease features. The efficacy benefit of epcoritamab was sustained with a predictable and manageable safety profile that was consistent with prior studies, and no new or long-term safety signals were identified. The capacity for outpatient administration of epcoritamab further supports its accessibility and convenience for patients with R/R FL in Japan. Overall, these data support the ongoing global phase 3 trials of epcoritamab plus rituximab and lenalidomide in second-line or later (FL-1; NCT05409066) and treatment-naive FL (FL-2; NCT06191744).

## Supplementary Information

Below is the link to the electronic supplementary material.

Supplementary file1 (DOCX 33 KB)

## Data Availability

Deidentified individual participant data collected during the trial will not be available upon request for further analyses by external independent researchers. Aggregated clinical trial data from the trial are provided via publicly accessible study registries/databases as required by law. For more information, please contact ClinicalTrials@genmab.com.
