# Supplementary material for: Three-year follow-up of epcoritamab therapy in Japanese patients with relapsed/refractory follicular lymphoma in EPCORE NHL-3
Source: Int J Hematol. 2026 Jan 18;123(5):696–707. doi: 10.1007/s12185-025-04139-1 (PMC13171670; doi:10.1007/s12185-025-04139-1)
Supplement: Supplementary file 1 — Supplementary file1 (DOCX 33 KB) [file 12185_2025_4139_MOESM1_ESM.docx]

**SUPPLEMENTARY APPENDIX: Three-year follow-up of epcoritamab therapy in Japanese patients with relapsed/refractory follicular lymphoma in EPCORE NHL-3**

Koji Izutsu, et al.

**Corresponding author:**

Koji Izutsu

Chief, Department of Hematology

National Cancer Center Hospital

Tsukiji 5-1-1

Chuo-ku, Tokyo 104-0045, Japan

Phone: +81 3 3542 2511

Fax: +81 3 3542 3815

Email: [kizutsu@ncc.go.jp](mailto:)

**Supplemental tables**

**Table S1.** Baseline demographic and disease characteristics in the overall R/R FL population

| Characteristic | Patients  (*N* = 21) |
| --- | --- |
| Median age, years (range) | 65 (58–75) |
| Sex at birth, *n* (%) |  |
| Male | 11 (52.4) |
| Ann Arbor stage, *n* (%) |  |
| I/II | 4 (19.0) |
| III | 7 (33.3) |
| IV | 10 (47.6) |
| FLIPI score, *n* (%) |  |
| 0/1 | 3 (14.3) |
| 2 | 7 (33.3) |
| 3–5 | 11 (52.4) |
| Bulky disease by IRC, *n* (%) |  |
| ≤ 6 cm | 19 (90.5) |
| > 6 cm | 2 (9.5) |
| Median time from diagnosis to first dose, years (range) | 8.4 (2–20) |
| Median time from end of last LOT to first dose, months (range) | 10.8 (2–68) |
| Median number of prior LOTs^a^ (range) | 4 (2–10) |
| ≥ 3 prior LOTs, *n* (%) | 13 (61.9) |
| ≥ 4 prior LOTs, *n* (%) | 11 (52.4) |
| Prior ASCT, *n* (%) | 4 (19.0) |
| Relapsed ≤ 12 months after ASCT, *n*/*N* (%) | 1/4 (25.0) |
| POD24 after first-line CIT, *n* (%) | 10 (47.6) |
| POD24 after any first-line therapy, *n* (%) | 12 (57.1) |
| Prior CAR T-cell therapy, *n* (%) | 0 |
| Double refractory,^b,c^ *n* (%) | 12 (57.1) |
| Primary refractory,^b,d^ *n* (%) | 6 (28.6) |
| Refractory^b^ to last prior systemic therapy, *n* (%) | 10 (47.6) |
| Prior bendamustine treatment, *n* (%) | 17 (81.0) |

^a^All patients had prior treatment with an anti-CD20 mAb and an alkylating agent. Other prior systemic treatments included bendamustine (81.0%), anthracyclines (76.2%), topoisomerase inhibitors (33.3%), nucleotides (28.6%), lenalidomide (23.8%), PI3K inhibitors (14.3%), and BCL-2 inhibitors (9.5%)

^b^No response or relapse within 6 months after therapy

^c^Refractory to both an anti-CD20 mAb and an alkylating agent

^d^Refractory to first-line therapy

*ASCT* autologous stem cell transplant, *BCL-2* B-cell lymphoma 2, *CAR* chimeric antigen receptor, *CIT* chemoimmunotherapy, *FLIPI* Follicular Lymphoma International Prognostic Index, *IRC* independent review committee, *LOT* line of treatment, *mAb* monoclonal antibody, *PI3K* phosphoinositide 3-kinase, *POD24* progression of disease within 24 months, *R/R FL* relapsed/refractory follicular lymphoma
